# Unveiling a Silent Bone Lesion: Pathological Fracture in a Child With Non‐Ossifying Fibroma

**DOI:** 10.1002/ccr3.72742

**Published:** 2026-05-18

**Authors:** Abu Omayer, Hasan A. BaniHani, Fathimathul Henna, Hamza Alshareh, Moussa Nassar, Marc Najjar

**Affiliations:** ^1^ Dr. Sulaiman Al Habib Hospital Dubai UAE; ^2^ University of Sharjah Sharjah UAE; ^3^ Dubai Medical College for Girls Dubai UAE; ^4^ Gilbert and Rose‐Marie Chagoury School of Medicine Lebanese American University Byblos Lebanon

**Keywords:** distal femur lesion, non‐ossifying fibroma, open reduction and internal fixation (ORIF), pathological fracture, pediatric orthopedic surgery

## Abstract

Non‐ossifying fibroma (NOF) is a benign, self‐limiting fibrous bone lesion commonly seen in children and adolescents, generally affecting the metaphysis of long bones. Although typically asymptomatic, larger NOF lesions can predispose patients to pathological fractures, especially in weight‐bearing bones. An 11‐year‐old boy presented to the emergency room following a minor knee trauma that caused pain and an inability to bear weight on the affected leg. Imaging studies identified a large, well‐defined lytic lesion in the distal femur, primarily suspected to be an aneurysmal or simple bone cyst with a related pathological fracture. Surgical management included open reduction and internal fixation (ORIF) of the fracture, curettage of the lesion, and bone grafting. Histopathological analysis confirmed the lesion as an NOF, demonstrating typical cellular patterns and evidence of fracture‐associated changes. In conclusion, this case highlights the importance of considering NOF in pediatric patients with fractures following minor trauma. Recognition of NOF and prompt intervention, as illustrated here, are necessary for effective treatment, particularly in large lesions within weight‐bearing bones.

**Key Clinical Message**

Non‐ossifying fibroma (NOF) is a common benign childhood lesion that often resolves spontaneously. Large NOFs in weight‐bearing bones may fracture after minor trauma. Consider NOF in children unable to bear weight after low‐impact injury. Radiographs are usually diagnostic; histology confirms complicated cases. ORIF with curettage and grafting can restore function.

## 1 Introduction

Non‐ossifying fibroma is the most common benign bone tumor in children. The exact incidence of NOFs is unknown; however, imaging studies estimate that up to 30% of children may have one or more undetected lesions, and the male population is disproportionately affected [1]. Plain radiographs are often sufficient to detect the lesion, except in cases of ambiguity, when an MRI may be necessary [1, 2]. It most commonly affects the metaphysis of long bones, but is not limited to this area. The prognosis is excellent: lesions are typically self‐limiting and commonly disappear by age 20–25 years as skeletal maturity is reached [2]. Therefore, a biopsy is not routinely required to confirm the diagnosis of NOF when characteristic clinical–radiographic features are present; however, histopathological confirmation may be necessary in indeterminate cases with ill‐defined margins, aggressive periosteal reaction, or a soft‐tissue component, or when lesions are surgically managed due to complications such as pathological fracture [3]. When obtained, histology typically shows spindle‐shaped fibroblasts, multinucleated giant cells, and foamy histiocytes [4].

## 2 Case History/Examination

An 11‐year‐old boy presented to the emergency department with an inability to bear weight on his right lower limb following a fall from monkey bars. The mechanism of injury involved axial loading after landing on the ground, resulting in acute right knee pain. On examination, the patient was conscious, hemodynamically stable, and oriented. Local examination revealed swelling and tenderness around the right knee without neurovascular compromise. There was no significant past medical or family history. Laboratory investigations were within normal limits.

## 3 Differential Diagnosis, Investigations, and Treatment

Plain radiographs of the right femur revealed a well‐defined, lytic, multiloculated lesion measuring approximately 11.8 cm in length and 4.1 cm in width, located in the distal diaphysis‐metaphysis region of the femur. The lesion had a scalloped, multiseptated appearance and was associated with a minimally displaced pathological fracture along the posterior‐medial cortex. There was no evidence of physeal involvement, periosteal reaction, extraosseous soft tissue extension, or matrix mineralization. Differential diagnosis included a NOF, aneurysmal bone cyst (ABC), or simple bone cyst (SBC), with pathological fracture.

**FIGURE 1:**
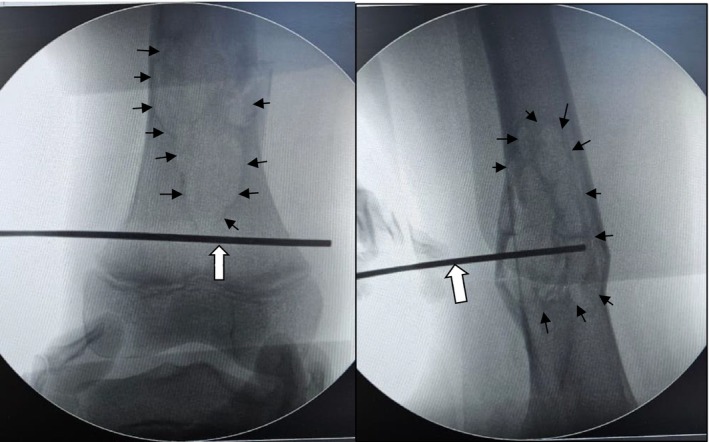
Intraoperative fluoroscopic frontal view showing K‐wire localization and lesion margins in the distal right femur. The K‐wire (white arrow) is used to localize the planned incision/entry point under fluoroscopy. The non‐ossifying fibroma (NOF) cavity is outlined by black arrows, indicating the area targeted for fluoroscopy‐guided curettage.

**FIGURE 2:**
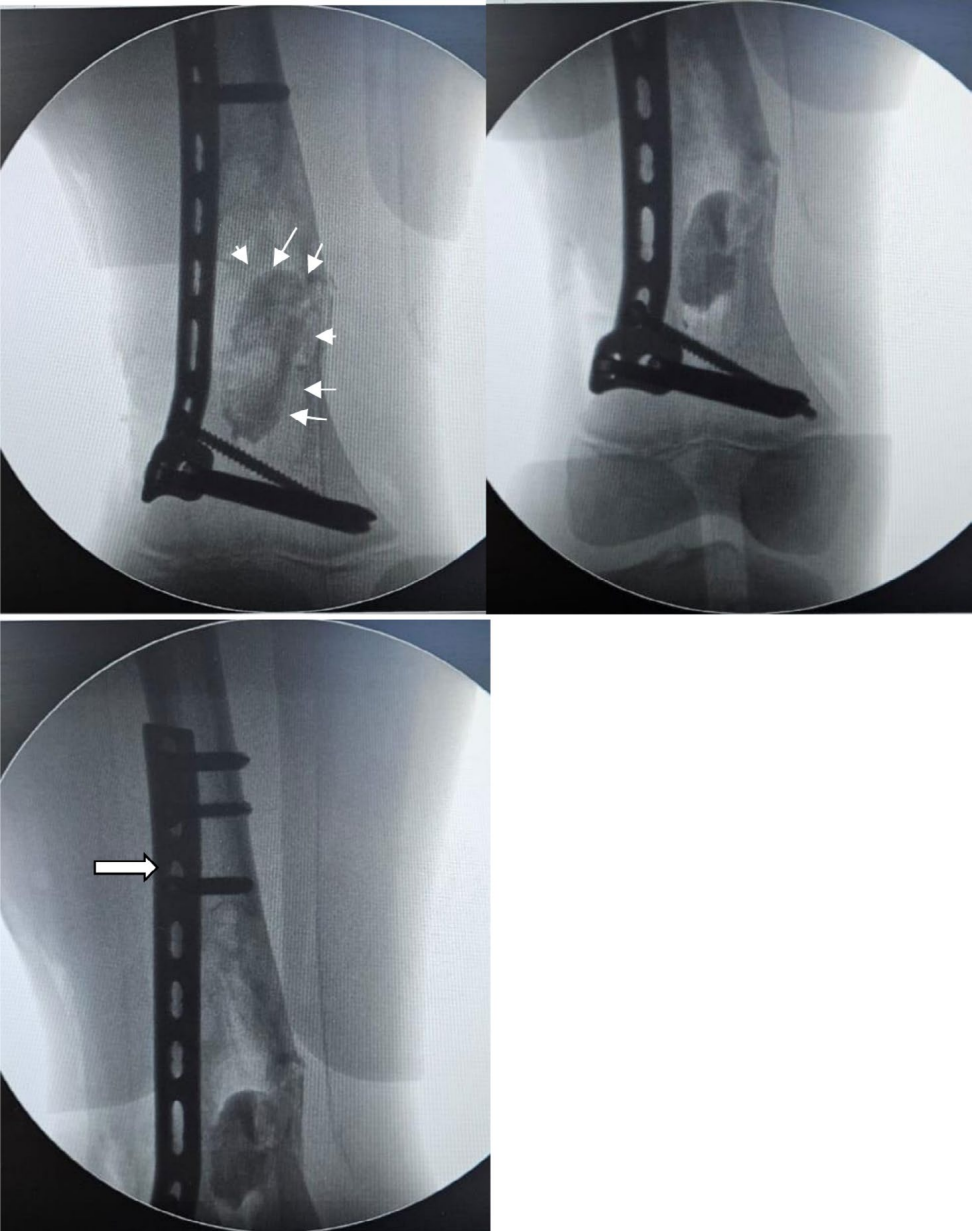
Intraoperative fluoroscopic frontal views demonstrating fixation, defect filling, and implant position in the distal right femur. The plate‐and‐screw construct is shown in satisfactory position with maintained alignment of the distal femur. The curetted lesion cavity is seen filled with bone graft (white arrows, left panel). The fixation hardware is intact and appropriately seated (white arrow, right panel).

The patient was admitted for elective open reduction and internal fixation of the distal femoral fracture, along with curettage of the lesion and bone grafting. Surgery was performed on the third day post‐admission. Under fluoroscopic guidance, the lesion was accessed, and intralesional curettage was performed. Approximately 5 cc of 1–4 mm‐sized cancellous bone chips were used to fill the defect. Internal fixation was carried out using a 152 mm, 8‐hole 4.5 mm locking compression T‐plate, secured with appropriate cortical and locking screws (Figure 1 and Figure 2).

**FIGURE 3:**
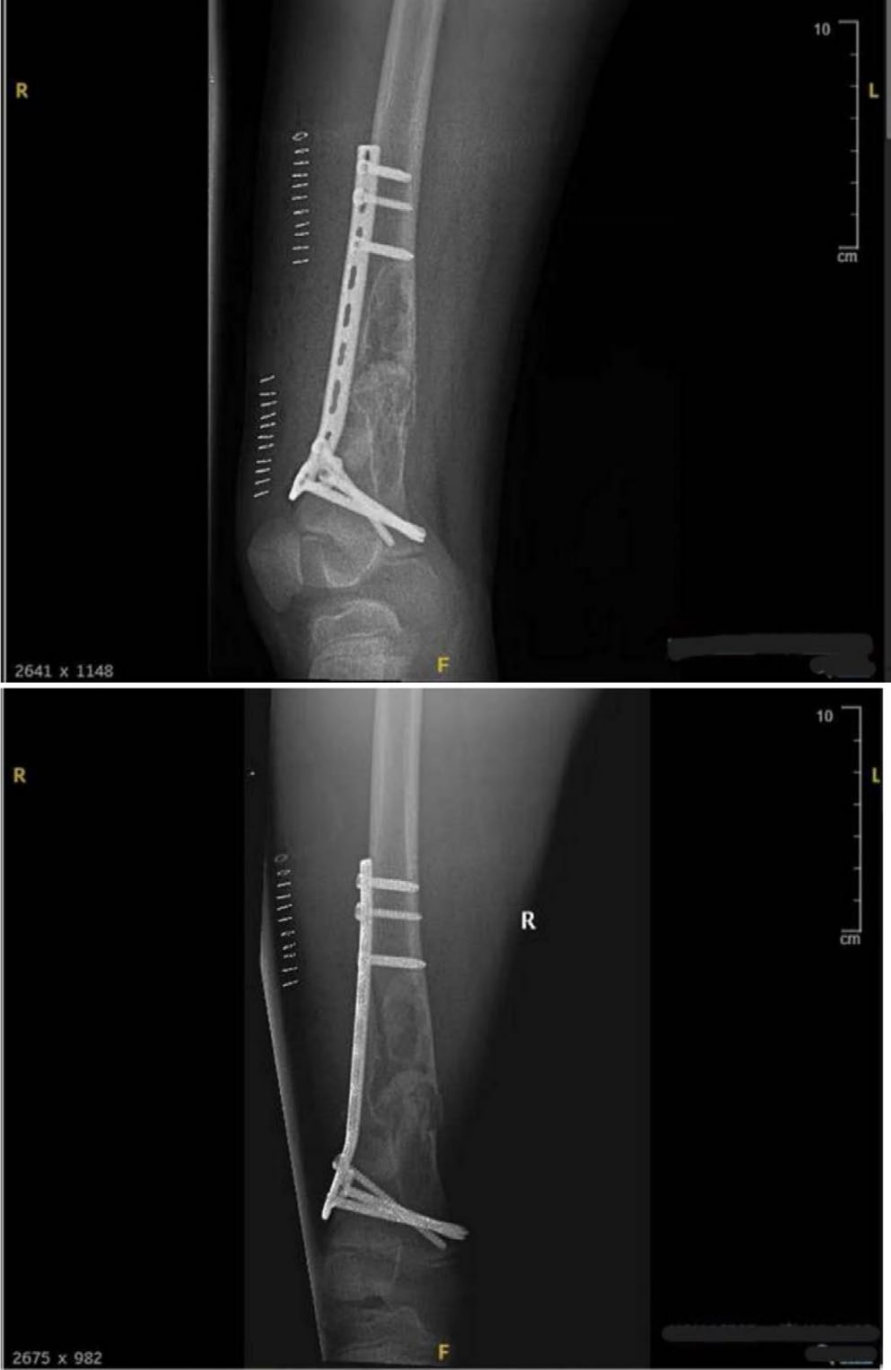
Early postoperative anteroposterior and lateral radiographs showing distal femoral fixation and grafted curettage defect. The distal femoral pathological fracture demonstrates maintained alignment after ORIF with plate‐and‐screw fixation and bone grafting of the curetted lesion cavity. No implant malposition is seen. Technical note: The lateral view is suboptimal due to pain‐limited positioning.

Postoperatively, the patient remained stable and tolerated oral intake. Initial pain management and physical therapy were initiated; however, ambulation was delayed due to pain and parental concerns. On postoperative day 4, the patient was mobilized with elbow crutches and began non‐weight‐bearing physiotherapy. An anteroposterior and lateral radiograph of the right femur using a long cassette was obtained. The radiology report described a bone cyst in the distal right femur with a pathological fracture that had been treated with bone grafting, plates, and screws. The bone fragments were noted to be properly aligned (Figure 3). The patient was subsequently discharged with instructions for continued rehabilitation and scheduled outpatient follow‐up.

**FIGURE 4:**
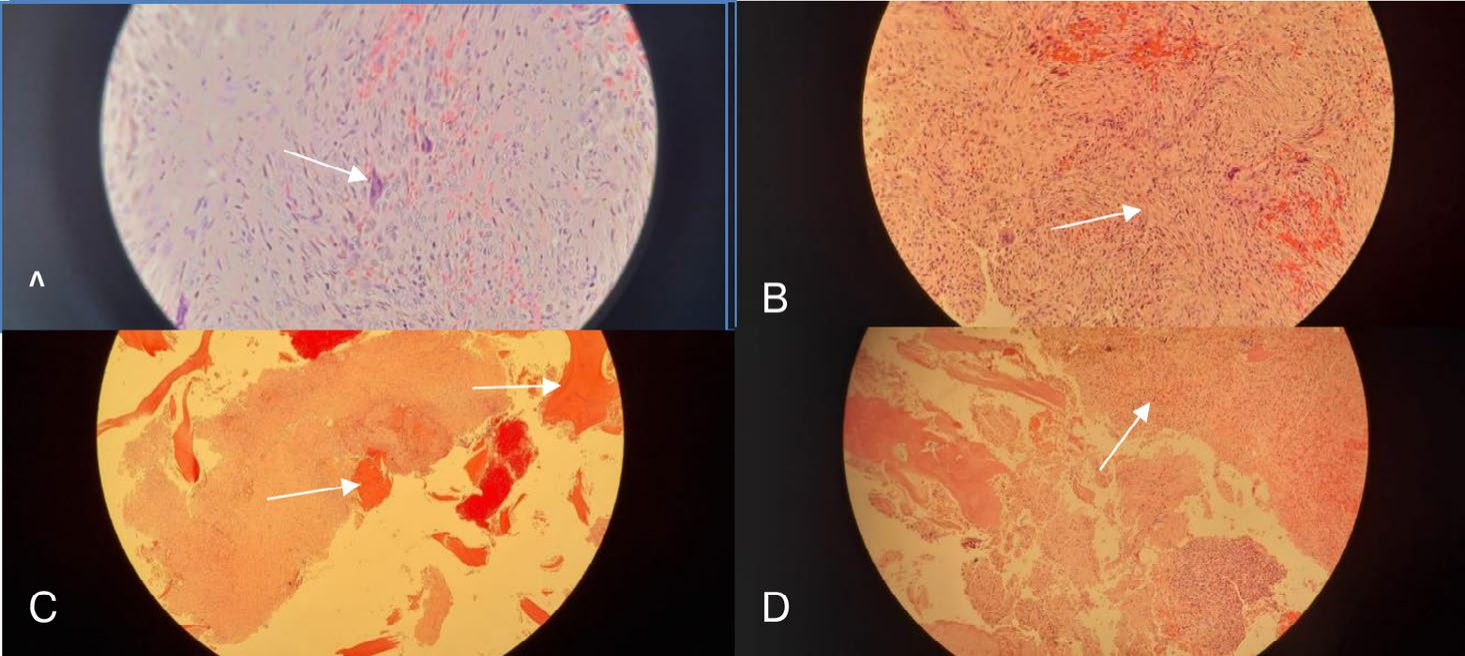
Histopathology confirming non‐ossifying fibroma (NOF) with fracture‐related reactive changes. (A) Spindle fibro‐histiocytic stroma in a storiform pattern. (B) Scattered multinucleated giant cells within the lesional stroma. (C) Fragmented cortical bone. (D) Reactive fibrocellular tissue and hemorrhagic/reparative changes consistent with recent fracture. White arrows highlight the referenced microscopic features in each panel.

Histopathological evaluation of the curetted specimen, available 9 days postoperatively, revealed multiple fragments of mildly cellular, fibro‐histiocytic spindle cells arranged in a storiform pattern (Figure 4 [A]). Scattered multinucleated giant cells and hemosiderin‐laden macrophages were present (Figure 4 [B]). No matrix production was observed. Additional fragments showed reparative fibrous tissue, necrotic debris, and fragmented cortical bone, consistent with recent fracture changes (Figure 4 [C and D]). Based on the clinical and histologic correlation, a diagnosis of NOF complicated by pathological fracture was made.

## 4 Conclusion and Results (Outcome and Follow‐Up)

**FIGURE 5:**
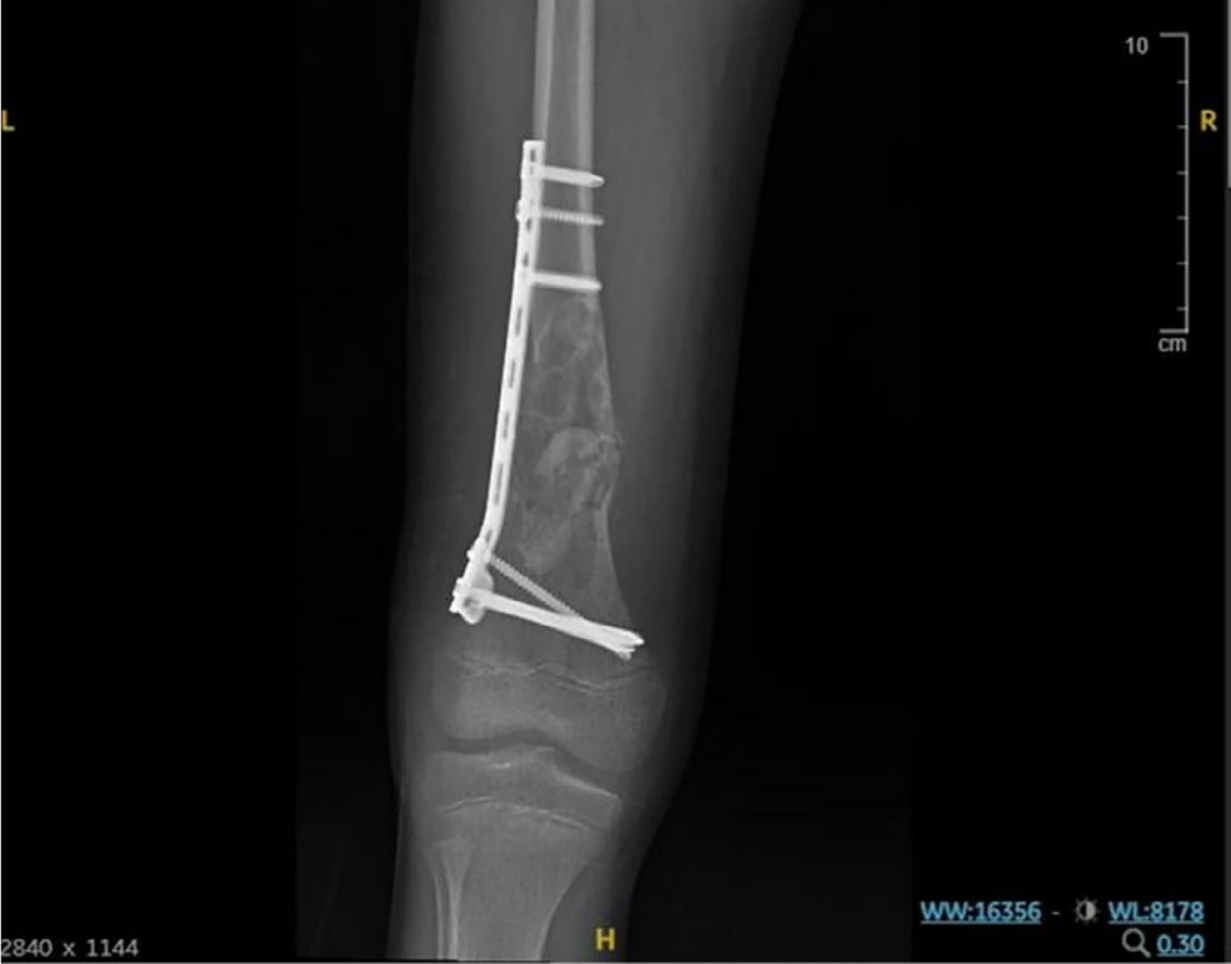
Postoperative anteroposterior radiograph showing intact distal femoral fixation with preserved physis and knee joint space. Plate‐and‐screw fixation remains in satisfactory position with maintained alignment and no evidence of implant failure. The distal femoral epiphysis and physis are intact, and the knee joint space is preserved without radiographic signs of effusion.

**FIGURE 6:**
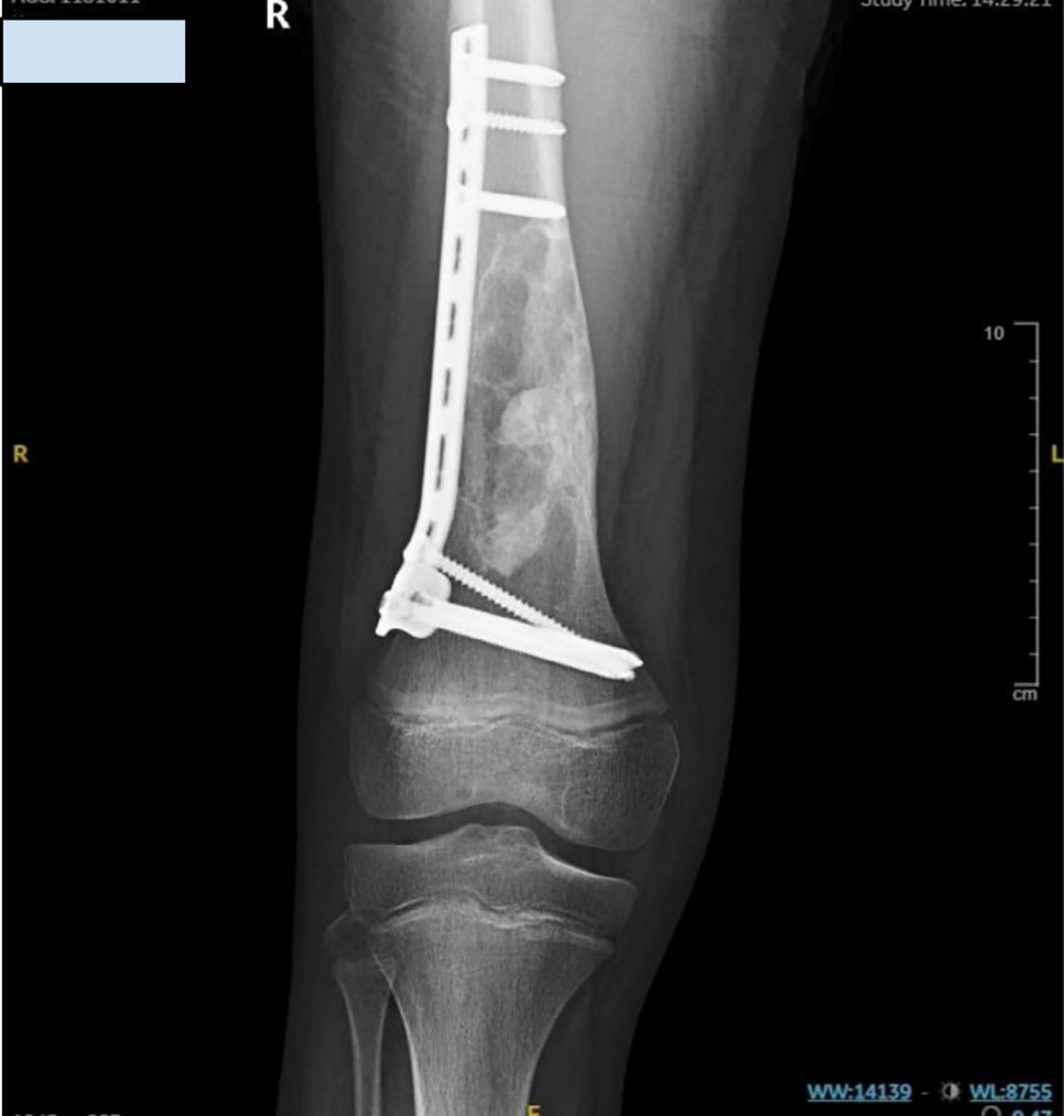
Four‐month follow‐up anteroposterior radiograph showing interval healing and graft incorporation after ORIF. There is progressive osseous remodeling with incorporation of graft material within the prior curettage defect. Alignment is maintained, fixation hardware remains intact, and there are no radiographic signs of implant loosening or recurrent lesion.

**FIGURE 7:**
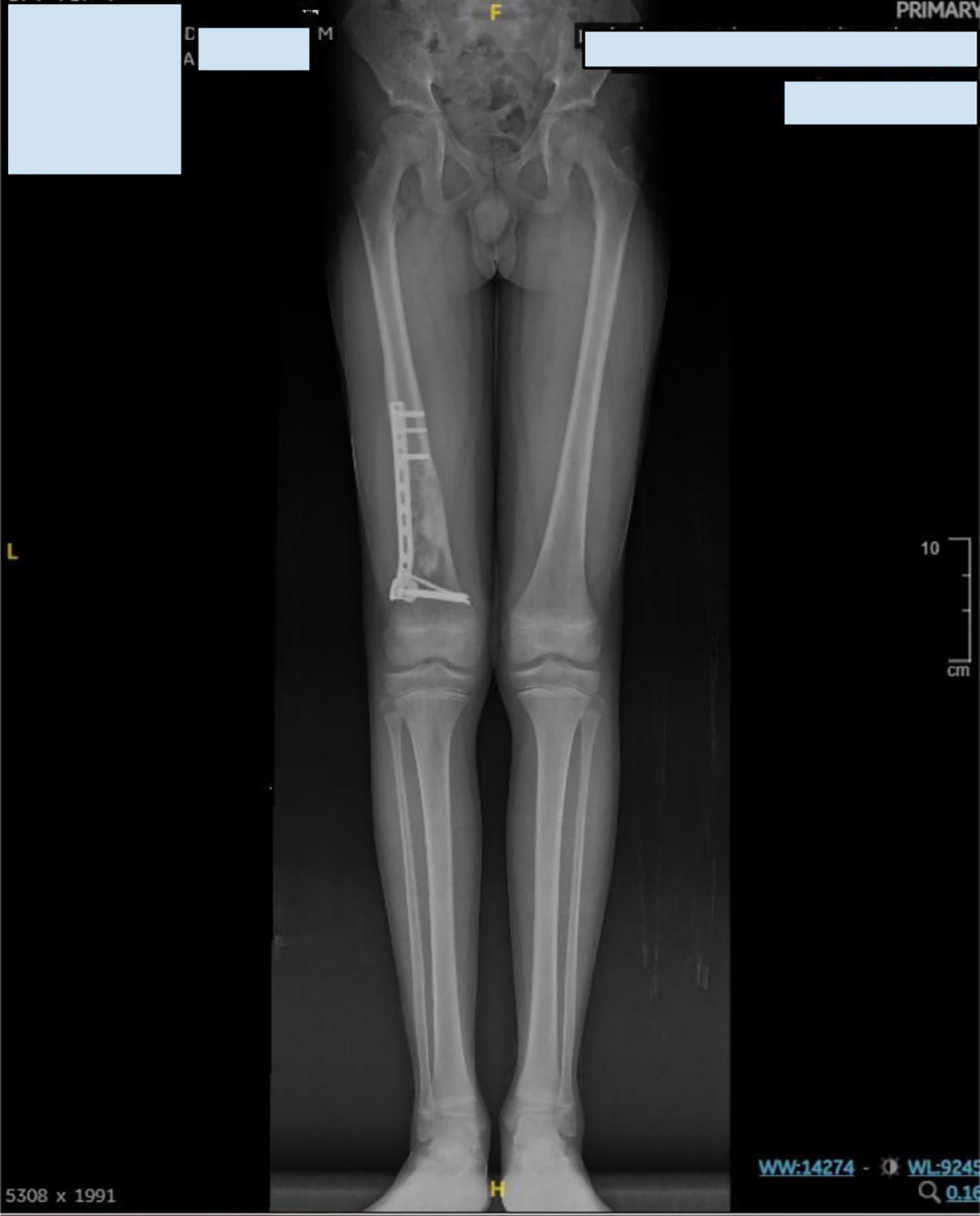
Nine‐month follow‐up standing long‐leg radiograph demonstrating complete healing and equal limb length. The distal femur shows complete radiographic healing with maintained alignment and an intact plate‐and‐screw construct. Comparison of both lower limbs demonstrates no clinically significant leg‐length discrepancy.

At the postoperative follow‐up on day 19, the patient presented for evaluation after sustaining a minor fall at school during physical activity. Radiographs were repeated, which showed maintained alignment of the fixation construct and no signs of implant failure or recurrent lesion (Figure 5). At the 4‐month follow‐up, the patient reported being pain‐free and was able to walk and run independently, though he avoided jumping activities (Figure 6). At 9 months, he had resumed participation in nonaggressive sports without complaints (Figure 7). He is scheduled for further review in 3 months to assess the possibility of hardware removal.

This case report highlights the importance of identifying NOF as a possible cause of pathological fractures in pediatric patients, even following minor trauma. The large size and location of the lesion in a weight‐bearing bone notably increased the risk of fracture, demanding surgical intervention for stabilization and lesion management. Critical insights from this case include the importance of combining radiological and histopathological evaluation for precise diagnosis, as well as the effectiveness of prompt surgical procedures, such as ORIF, curettage, and bone grafting, in achieving favorable outcomes. Clinicians should keep a high index of suspicion for NOF in children presenting with localized pain, swelling, and fractures after minimal injury, specifically in the metaphyseal regions of long bones. Future research should aim to optimize risk assessment for pathological fractures in NOF cases and investigate molecular mechanisms underlying lesion progression to inform potential nonsurgical treatment strategies. Early recognition and tailored management remain essential to prevent complications and ensure the most favorable recovery.

## 5 Discussion

This case highlights a rare presentation of a NOF complicated by a pathological fracture in an 11‐year‐old boy. The patient presented with localized pain, swelling, and inability to bear weight following minor trauma to the distal femur. Radiographic evaluation revealed a large, well‐circumscribed lytic lesion with scalloped margins, initially suggestive of a simple bone cyst or aneurysmal bone cyst. Definitive diagnosis of NOF was established through histopathological examination. Given the lesion's considerable size and its contribution to structural compromise, surgical management was undertaken, including open reduction and internal fixation (ORIF), intralesional curettage, and bone grafting to stabilize the fracture and manage the underlying pathology.

While MRI is often considered valuable for preoperative assessment, especially when conventional radiographs suggest aggressive bone lesions or neoplasia, it was not deemed necessary in our case. In most patients, NOF can be confidently diagnosed on plain radiographs, and MRI is generally reserved for lesions with atypical features or when local staging is required [5]. In our patient, the radiographs demonstrated a well‐defined, localized lytic lesion without aggressive periosteal reaction or soft‐tissue component, and the acute symptoms were explained by the pathological fracture, supporting a radiographic‐based approach [3]. When MRI is indicated in the evaluation of suspected bone tumors, it should ideally be obtained before biopsy‐related bleeding or swelling, which can complicate interpretation and staging. Importantly, MRI can be misleading in the setting of an acute or healing fracture: cystic lesions may be difficult to characterize on plain radiographs in some cases, and post‐fracture MRI can show heterogeneous fluid signal and peripheral or nodular enhancement related to hemorrhage and early reparative change rather than true lesion aggressiveness [6]. Given that fixation was required regardless of advanced imaging, and that histopathology obtained during curettage provides definitive diagnosis in complicated presentations, proceeding directly with ORIF, curettage, and biopsy was reasonable and consistent with the stepwise diagnostic strategy [7].

This approach is supported by prior reports emphasizing the need to correlate imaging with histology in fracture‐complicated lesions. Hoeffel et al. described pediatric cases in which fractures through NOFs altered histologic appearances and initially led to misdiagnosis as aneurysmal bone cysts, with radiologic reevaluation ultimately confirming NOF, highlighting the risk of diagnostic pitfalls when fracture‐related changes are present [8]. Rammanohar et al. also reported that typical NOF morphology on plain radiographs was sufficient for diagnosis in most cases, yet MRI was frequently requested as additional investigation, underscoring the importance of relying on characteristic radiographic features and reserving MRI for truly atypical or indeterminate presentations [3].

Although the volume of graft used was small (5 cc of 1–4 mm cancellous chips), it was considered sufficient because the distal femoral locking plate provided definitive biomechanical stability. Locking plates with fixed‐angle screws function as a fixed‐angle, single‐beam construct that improves stability under axial load and limits plate‐to‐bone friction, thereby helping preserve periosteal blood supply [9]. In this setting, the graft's role was primarily to fill the post‐curettage defect and provide osteoconductive support to facilitate healing, rather than to serve as structural reinforcement [10]. In a skeletally immature patient, remodeling potential depends on remaining growth and proximity to an active physis; therefore, fixation was positioned to protect the physis and minimize the risk of growth disturbance [9]. Autologous bone grafting was avoided due to its well‐documented drawbacks, including the need for a second surgical site, increased operative time, and potential donor site morbidity such as infection, hematoma, and chronic pain. Additionally, harvesting autograft in a pediatric patient presents technical and ethical challenges, especially when effective synthetic alternatives are available [11, 12].

Postoperative rehabilitation after ORIF and grafting for a distal femoral fracture can follow a stepwise physiotherapy approach similar to protocols used after ORIF for femoral fractures, with progression guided by pain, function, and radiographic healing. An early phase emphasizes ankle range of motion and quadriceps and gluteal isometrics, followed by active and active‐assisted hip, knee, and ankle exercises, initiation of non‐weight‐bearing ambulation with an assistive device, and later staged progression to partial and then full weight bearing [13]. This staged progression is consistent with the time course reported in distal femoral NOF‐related fractures, where initial avoidance of weight bearing with crutches is followed by progressive weight bearing over several weeks and return to jogging within a few months once pain resolves and gait normalizes [2]. Given the initial delay in ambulation in our patient, structured parental reassurance and patient and family education were emphasized to reduce fear‐avoidance and support engagement with physiotherapy. In our case, this approach was associated with excellent functional recovery, with the patient walking and running independently by the 4‐month follow‐up.

Although lesion size is often cited as a predictor of pathological fracture, the literature shows that size alone is an imperfect surrogate for mechanical failure. In the Mayo Clinic series by Arata et al., fractures occurred when lesions occupied more than 50% of the bone diameter on both views and exceeded 33 mm in length, emphasizing the importance of relative lesion size and cortical involvement rather than absolute dimensions alone [14]. Herget et al. similarly linked fracture risk to substantial lesion expansion relative to bone diameter, reflecting progressive cortical thinning and loss of structural integrity [1].

In contrast, Easley and Kneisl found that most “large” NOFs in weight‐bearing bones did not fracture despite exceeding size thresholds, arguing against prophylactic surgery based on size alone [15]. Sakamoto et al. similarly observed that many large lower‐extremity lesions are clinically silent, whereas upper‐extremity lesions, particularly in the radius, may be more prone to fracture due to slender‐bone biomechanics [16]. Mankin et al. additionally reported that fracture risk and surgical requirement are higher in syndromic forms such as Jaffe–Campanacci syndrome, which features multiple NOFs with associated clinical findings [13]. Taken together, these findings suggest that fracture risk depends on lesion location, biomechanical load, and the degree of cortical thinning and expansion rather than on size alone. In our patient, the lesion was extensive in a high‐load region of the distal femur and was associated with cortical compromise, which likely lowered the threshold for fracture after axial loading trauma.

Ritschl classified NOF into four radiologic stages. Stage A describes a small lesion adjacent to the growth plate. In Stage B, the lesion becomes “grape‐shaped” with thin sclerotic margins and lies farther from the growth plate. Stage C is characterized by increasing sclerosis with mineralization beginning from the diaphyseal side and progressing toward the growth plate, and Stage D represents complete homogeneous sclerosis. Stage B has been associated with the highest fracture risk. In the series by Herget et al., lesions were distributed as 19 in stage A, 53 in stage B, 17 in stage C, and 14 in stage D, with most detected incidentally; among 10 symptomatic patients, 6 (mean age 10 years) sustained pathological fractures, and 4 of these occurred in the tibia, all in stage B lesions. Stage B lesions that fractured showed marked expansion in both transverse and sagittal planes, and this stage persisted for an average of 21 months. The lesion in our case is most consistent with Stage B, given its size, cortical thinning, and presentation with pathological fracture following minor trauma [1].

The exact cause of NOFs remains unclear, but an association with tendinous structures has been proposed. Muzykewicz et al. reviewed 68 NOFs in 60 patients and found that most distal femoral lesions were located medially (60.3%) or laterally (36.7%). On CT, 93% of lesions demonstrated a relationship to adjacent tendon origins, most commonly the medial gastrocnemius (53.7%), followed by the lateral gastrocnemius (29.3%) and adductor magnus (9.8%); MRI in a small subset similarly suggested attachment to the medial gastrocnemius. These findings support the concept that NOFs may arise near the physis or metaphysis and appear to migrate with growth [8]. Our case is consistent with this proposed tendon‐origin predilection in the distal femur.

Although no genetic testing was performed in our patient, Baumhoer et al. recently reported that somatic mutations in KRAS, FGFR1, and NF1 activate the RAS‐MAPK pathway in a subset of NOFs, with mutations present in 64% and 14% of cases, respectively. These findings support classifying NOFs as neoplasms rather than reactive lesions, linking them to the broader RASopathy family of tumors. The study also observed estrogen receptor expression in some tumor cells, suggesting a potential link between puberty‐related estrogen signaling and the regression of NOFs [16]. This spontaneous regression, common in adolescents, is likely influenced by the balance between mutant and wild‐type cells. These results provide a deeper understanding of the molecular mechanisms driving NOFs and suggest further studies on the interplay between mutant and normal cells, as well as the role of estrogen, to improve treatment strategies for similar RAS‐MAPK‐activated tumors [15, 16].

## Author Contributions

**Abu Omayer:** conceptualization, investigation, project administration, visualization, writing – original draft, writing – review and editing. **Hasan A. BaniHani:** writing – original draft, writing – review and editing. **Fathimathul Henna:** writing – original draft, writing – review and editing. **Hamza Alshareh:** data curation, writing – review and editing. **Moussa Nassar:** writing – review and editing. **Marc Najjar:** supervision, validation.

## Funding

The authors have nothing to report.

## Ethics Statement

Ethical approval was obtained from the relevant institutional review board, and patient consent was acquired for the publication of this case.

## Consent

Written Consent obtained from the patient's guardian (father).

## Conflicts of Interest

The authors declare no conflicts of interest.

## Data Availability

Data is available upon request from the corresponding author.

## References

[1] G. W. Herget , D. Mauer , T. Krauß , et al., “Non‐Ossifying Fibroma: Natural History With an Emphasis on a Stage‐Related Growth, Fracture Risk and the Need for Follow‐Up,” BMC Musculoskeletal Disorders 17, no. 147 (2016), 10.1186/s12891-016-1004-0. PMC4820930 27044378

[2] A. Sakamoto , K. Tanaka , T. Yoshida , and Y. Iwamoto , “Nonossifying Fibroma Accompanied by Pathological Fracture in a 12‐Year‐Old Runner,” Journal of Orthopaedic & Sports Physical Therapy 38, no. 7 (2008): 434–438, 10.2519/jospt.2008.2655. 18591758

[3] G. Iacobellis , A. Leggio , C. Salzillo , S. Lucà , R. Ortega‐Ruiz , and A. Marzullo , “Analysis and Historical Evolution of Paediatric Bone Tumours: The Importance of Early Diagnosis in the Detection of Childhood Skeletal Malignancies,” Cancers 17, no. 3 (2025): 451, https://pubmed.ncbi.nlm.nih.gov/39941818/. 39941818 10.3390/cancers17030451 PMC11816121

[4] R. Margau , P. Babyn , W. Cole , C. Smith , and F. Lee , “MR Imaging of Simple Bone Cysts in Children: Not So Simple,” Pediatric Radiology 30, no. 8 (2000): 551–557. 10993540 10.1007/s002470000258

[5] M. D. Kumar , R. Singh , R. Khiyani , and K. Kaur , “Evaluation of Results of Open Distal Femur Fractures With Primary Fixation and Antibiotic Impregnated Collagen,” Chinese Journal of Traumatology 22, no. 6 (2019): 328–332, https://www.sciencedirect.com/science/article/pii/S1008127519301804. 31753759 10.1016/j.cjtee.2019.08.005 PMC6921170

[6] M. Takamoto , M. Takechi , K. Ohta , et al., “Risk of Bacterial Contamination of Bone Harvesting Devices Used for Autogenous Bone Graft in Implant Surgery,” Head & Face Medicine 9, no. 1 (2013), 10.1186/1746-160X-9-3. PMC3598468 23311758

[7] R. Dimitriou , G. I. Mataliotakis , A. G. Angoules , N. K. Kanakaris , and P. V. Giannoudis , “Complications Following Autologous Bone Graft Harvesting From the Iliac Crest and Using the RIA: A Systematic Review,” Injury 42 (2011): 3–15. 10.1016/j.injury.2011.06.015 21704997

[8] D. A. Muzykewicz , A. Goldin , N. Lopreiato , et al., “Nonossifying Fibromas of the Distal Tibia: Possible Etiologic Relationship to the Interosseous Membrane,” Journal of Children's Orthopaedics 10, no. 4 (2016): 353–358, 10.1007/s11832-016-0745-5. PMC4940240 27259988

[9] A. Sakamoto , R. Arai , T. Okamoto , and S. Matsuda , “Non‐Ossifying Fibromas: Case Series, Including in Uncommon Upper Extremity Sites,” World Journal of Orthopedics 8, no. 7 (2017): 561, 10.5312/wjo.v8.i7.561. 28808627 PMC5534405

[10] J. Rammanohar , C. Zhang , A. Thahir , and M. Krkovic , “Imaging of Non‐Ossifying Fibromas: A Case Series,” Cureus 13, no. 3 (2021), 10.7759/cureus.14102. PMC8075760 33927919

[11] M. E. Easley and J. S. Kneisl , “Pathologic Fractures Through Nonossifying Fibromas: Is Prophylactic Treatment Warranted?,” Journal of Pediatric Orthopaedics 17, no. 6 (1997): 808–813, 10.1097/01241398-199711000-00021. 9591988

[12] K. Alshehri and A. A. Fadil , “Non‐Ossifying Fibroma Pathological Fracture in a Patient With Lactose Intolerance,” Cureus 13, no. 8 (2021), 10.7759/cureus.17225. PMC8442721 34540452

[13] H. J. Mankin , C. A. Trahan , G. Fondren , and C. J. Mankin , “Non‐Ossifying Fibroma, Fibrous Cortical Defect and Jaffe–Campanacci Syndrome: A Biologic and Clinical Review,” Musculoskeletal Surgery 93, no. 1 (2009): 1–7, 10.1007/s12306-009-0016-4. 19711155

[14] C. Hoeffel , M. Panuel , F. Plenat , L. Mainard , and J.‐C. Hoeffel , “Pathological Fracture in Non‐Ossifying Fibroma With Histological Features Simulating Aneurysmal Bone Cyst,” European Radiology 9, no. 4 (1999): 669–671, 10.1007/s003300050730. 10354882

[15] J. V. Bovée and P. C. Hogendoorn , “Non‐Ossifying Fibroma: A RAS‐MAPK Driven Benign Bone Neoplasm,” Journal of Pathology 248, no. 2 (2019): 127–130, 10.1002/path.5259. 30809793 PMC6593856

[16] D. Baumhoer , M. Kovac , J. Sperveslage , et al., “Activating Mutations in the MAP‐Kinase Pathway Define Non‐Ossifying Fibroma of Bone,” Journal of Pathology 248, no. 1 (2019): 116–122. 30549028 10.1002/path.5216

